# Impact of COVID-19 lockdown on match performances in the National Basketball Association

**DOI:** 10.3389/fpsyg.2022.951779

**Published:** 2022-11-22

**Authors:** Peng Lu, Shaoliang Zhang, Jie Ding, Xing Wang, Miguel Angel Gomez

**Affiliations:** ^1^Division of Sport Science and Physical Education, Tsinghua University, Beijing, China; ^2^Facultad de Ciencias de la Actividad Física y del Deporte (INEF), Universidad Politécnica de Madrid, Madrid, Spain

**Keywords:** team sports, basketball performance analysis, match-related statistics, National Basketball Association, COVID-19

## Abstract

This study aimed to compare differences in the match performances between home and away games during pre- and post-COVID-19 lockdown and to identify the key factors to match success with and without spectators. The sample consisted of 1,549 basketball matches including 971 games of the 2019–2020 regular season before the COVID-19 lockdown and 578 ghost matches of the 2020–2021 regular season after the COVID-19 pandemic. The independent *t*-test was used to explore the differences before and after COVID-19 while univariate and multivariable logistic regression models were used to identify the key factors to match success between matches with and without spectators. Our study identified that offensive rebounds were the only indicator differentiating between home and away games after the COVID-19 lockdown. Furthermore, home teams won more matches than away matches before the COVID-19 whereas home advantage had no impact on winning matches after the COVID-19. Our study suggested that crowd support may play a key role in winning games in the NBA. Furthermore, independently of the pre-and post-COVID19 pandemic, free throws made, three-point field goals made, defensive rebounds, assists, steals, personal fouls, and opponent quality were key factors differentiating between win and loss. Coaches and coaching staff can make informed decisions and well prepare for basketball match strategies.

## Introduction

The consistently better performance seen by teams in various sporting contexts when playing at home is known as the “Home Advantage” (HA) that has a clear impact on winning basketball matches in the available research (Sampaio et al., 2006; Gómez and Pollard, 2011; García et al., 2014; Higgs, 2021; Mateus et al., 2021). Home teams have a better performance in terms of assists (García et al., 2014; Bustamante-sánchez et al., 2022), block shots (Sampaio et al., 2008; Garcia-Rubio et al., 2009; Bustamante-sánchez et al., 2022), and personal fouls (Sampaio et al., 2008; Bustamante-sánchez et al., 2022) than away teams. In particular, defensive rebounds seem to be the most common performance indicator that was influenced by HA in the NBA (Zhang et al., 2019b). Although research into the impact of HA on match performances during basketball match-play has produced equivocal results, it is one of the most important contextual variables that should be taken into consideration in basketball science.

Factors that affected the phenomenon have been paid constant attention over the past years (Nevill and Holder, 1999; Neave and Wolfson, 2003; Pollard, 2008; Gómez and Pollard, 2011; Ribeiro et al., 2016; Goumas, 2017; Ponzo and Scoppa, 2018; Fischer and Haucap, 2021; Tilp and Thaller, 2020). Crowd support, territoriality, familiarity with the stadium, and travel fatigue (Ponzo and Scoppa, 2018) are believed to be the key factors of the HA phenomenon. Furthermore, the available research showed that crowd support and density might be the two most important factors that contributed to the HA (Inan, 2020). Ponzo and Scoppa (2018) identified that HA leads to the improvement of team performance and biased decisions of referees. For example, Fioravanti et al. (2021) found that HA has dropped by approximately 5% and the point difference in favor of home teams was reduced from approximately 6 to 4 points on average when a ghost game took place in rugby competitions. Similarly, a study in professional basketball demonstrated that HA affects the microscopic dynamics of the game by increasing the scoring rates and decreasing the time intervals between scores (Ribeiro et al., 2016). However, there is still no common consensus about the relative importance and interactive impact of different factors (Wunderlich et al., 2021).

The lockdowns due to the Covid-19 pandemic provided a unique opportunity to test a natural experiment in terms of team performances that could be analyzed during matches with and without the presence of an audience (McCarrick et al., 2021). Tilp and Thaller (2020) reported that the Covid-19 lockdown caused a decreased trend for HA in Germany’s top football league, Bundesliga. Furthermore, this study pointed out that the ambiguity in previous studies’ findings may result from different ways of proxying home support (e.g., occupancy rate or absolute attendance) or various degrees of control for covariates (Tilp and Thaller, 2020). Also, the other study examined the impact of crowd support on match performances in the three German men’s professional football divisions, they found that there was a reduced HA in the first division in the ghost games, whereas no change was observed in the second and third divisions (Fischer and Haucap, 2021). Indeed, Arboix-Alió et al. (2022) reported that the effect of HA did not disappear despite playing without spectators but decreased from 63.99 to 57.41% while playing with spectators benefited local teams’ performance, especially in the Portuguese and Italian Hockey leagues (Arboix-Alió et al., 2022). To our knowledge, four studies have examined changes in HA due to the COVID-19 epidemic in the NBA where the presence of crowds was associated with rebounds and points differential and accounted for a 15.91% increase in terms of winning percentage in comparison with the absence of crowds (Leota et al., 2021; Alonso et al., 2022; Bustamante-sánchez et al., 2022; Gong, 2022; Szabó, 2022). However, these studies fail to consider the impact of situational variables and the variability from season to season. To increase the statistical power of the analysis and the accuracy of the outcomes, our study uses a larger sample size controlling for situational variables to explore the changes in HA before and after the COVID-19 pandemic.

The drivers and mechanisms of HA remain equivocal, yet HA is a robust and reliable phenomenon (Leota et al., 2021). Given the significance of understanding which team performances may be more affected by crowd support in professional basketball, the purpose of this study was to compare differences in the match performances during pre- and post-COVID-19 lockdown and to identify the key factors to winning matches with and without spectators. We hypothesized that there might be a decreased trend after COVID-19 compared to the period before the COVID-19.

## Materials and methods

### Sample

Data were collected from the official NBA website (and www.basketball-reference.com). An observational case series study design was used to compare match performances before and after COVID-19. A total of 1,549 basketball matches included 971 games of the 2019–2020 NBA regular season before the COVID-19 lockdown and 578 ghost matches of the 2020–2021 NBA regular season after the COVID-19 pandemic. Additionally, the game-related statistics included two-and three-point field goals (both made and missed), free-throws (both made and missed), defensive and offensive rebounds, assists, blocks, fouls, steals, turnovers, and personal fouls. Based on the previous research (Sampaio et al., 2006; García et al., 2014; Zhang et al., 2017), a total of 13 variables were selected to quantify the technical performances.

### Procedures

Furthermore, to control for the situational conditions during different matches, match location, opponent quality, and match type were considered in our study, and the detailed explanation is as follows:

- Match location: This was defined as the match being played at home or away (Gómez et al., 2010).
- Opponent quality: This was defined using the team’s winning match percentage (Gómez et al., 2013a). A k-means cluster analysis identified two clusters: weak teams (before the COVID-19 lockdown: winning = 37.3 ± 7.6%, after the COVID-19 lockdown: winning = 33.7 ± 7.5%) and strong teams (before the COVID-19 lockdown: winning = 66.4 ± 6.8%, after the COVID-19 lockdown: winning = 59 ± 7.6%).
- Match type: According to the previous studies (Zhang et al., 2019b), a k-means cluster analysis was performed on the entire sample with the aim of creating and describing maximal different groups of match type (balanced and unbalanced matches). The cubic clustering criterion, together with Monte Carlo simulations, was used to identify the optimal number of clusters, thereby avoiding using subjective criteria. A k-means cluster analysis identified a threshold for scoring differences of a match with balanced (cluster 1, 1–14 points difference) and unbalanced (cluster 2, >15 points difference) matches identified.

To control for game rhythm, all variables were then normalized according to game ball possessions and multiplied by 100 (Kubatko et al., 2007). Additionally, possessions are the most important value of advanced statistics, as they are the basis for comparing the indicators that are generated. All calculations such as offensive efficiency, defensive efficiency, rebounding rate or percentage of assists, and shooting accuracy, are normalized on the basis of possessions played. In this way we can compare different games or leagues in future studies. Briefly, ball possessions were calculated using the following equation: 0.976 × (field-goal attempts + (0.4 × free-throw attempts) – “offensive rebounds” + “turnovers”) (Kubatko et al., 2007). To assess the validity of data sets, a sub-sample of 10 games was randomly selected and observed by two experienced analysts (basketball coaches with more than 5 years of experience in basketball performance analysis) who recorded key performance indicators. First, two basketball experts were interviewed separately and answered the following question: “In your opinion, which information (technical and tactical actions) can we extract from the match is the more relevant current study?” The basketball experts have the following profiles: Expert 1 – professor in basketball science at a local university; Expert 2 – Assistant coach in a professional basketball club. Then, the experts’ answers were compiled and analyzed by the authors of this study (Santos et al., 2022). These results were contrasted with those gathered within the official website, and perfect Intra-class Correlation Coefficients (ICC = 1.0) were obtained for free-throws, two-and three-point field-goals (both made and missed), offensive and defensive rebounds, turnovers, steals, blocked shots, personal fouls. A lower but very acceptable (ICC = 0.93) was obtained for the final performance indicator, assists. All procedures were approved by the local Institutional Research Review Board.

### Statistical analysis

Data normality assumptions were verified by using the Kolmogorov–Smirnov test and homogeneity of variance was testified by the Levene test. Data were presented as mean ± standard deviation. An independent t-test was used to identify the difference in the game performance-related variables of the home and away teams before and after the COVID-19 epidemic. To clarify the meaningfulness, Cohen’s *d* effect sizes and 95% confidence intervals (CI) were calculated (Cohen, 1988; Fritz et al., 2012). Effect sizes (ES) were interpreted as follows: ≤0.2 trivial, >0.2–0.6 small, >0.6–1.2 moderate, >1.2–2.0 large, >2.0–4.0 very large, and > 4.0 extremely large (Hopkins et al., 2009).

Then, binary logistic regression model was used to identify the key winning factors for both 971 games of the 2019–2020 NBA regular season before the COVID-19 lockdown and 578 ghost games of the 2020–2021 NBA regular season after the COVID-19 pandemic, specifically. Univariate analysis was used to identify individual predictors. Variables with a univariate significance of *p* < 0.01 were entered into a multiple stepwise regression analysis to determine the independence of these predictors (Fairbairn et al., 2012). A significance level of *p* < 0.05 was considered statistically significant. All statistical analyses were performed in R (version 3.5.3; Boston, MA).

## Results

### The differences between home and away matches before and after COVID-19

**Figure 1:**
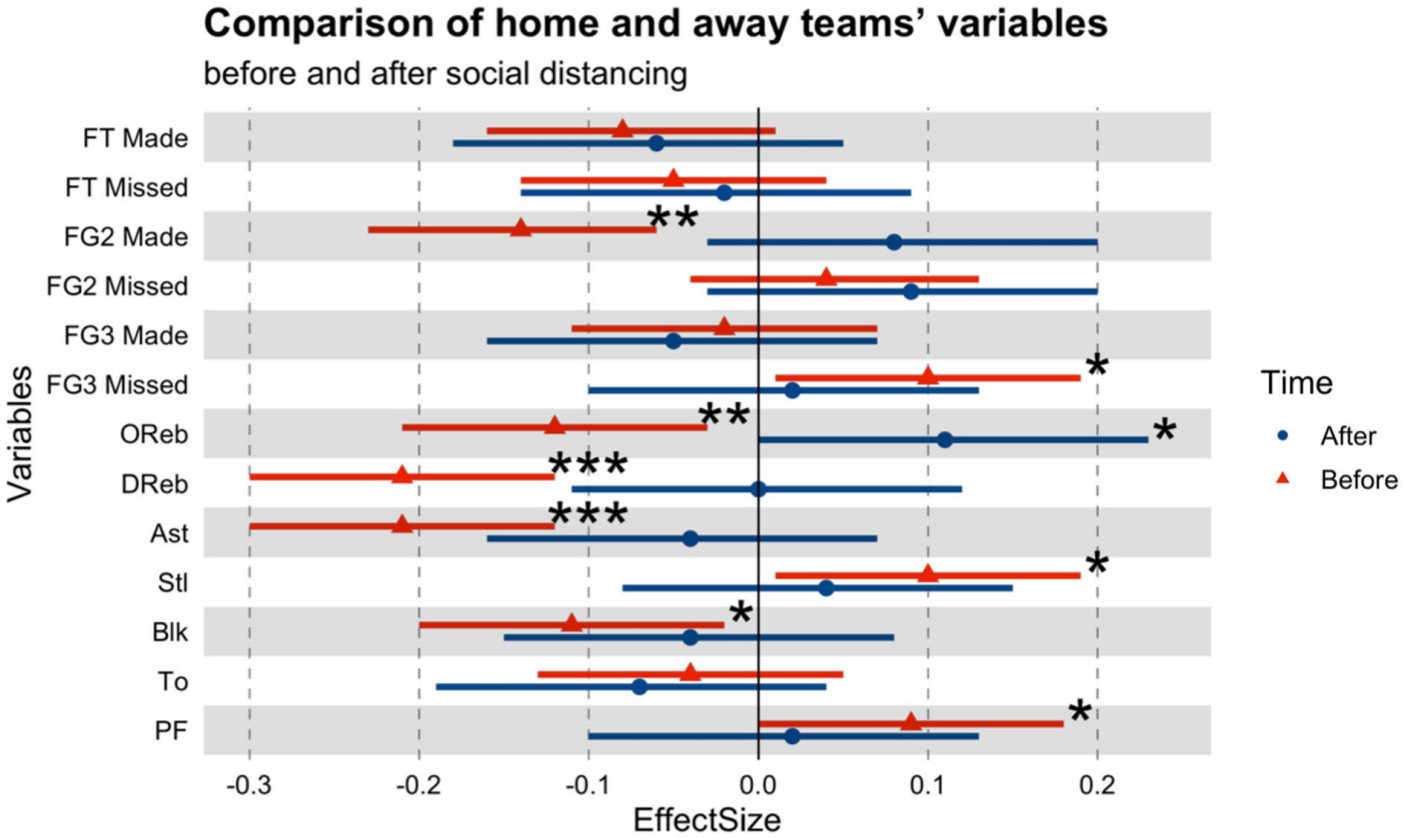
Comparison of home and away teams’ variables before and after social distancing. *p < 0.05, **p < 0.01, ***p < 0.001. FT, free-throw; FG2, two field-goals; FG3, three field-goals; OReb, offensive rebounds; DReb, defensive rebounds; Ast, assists; Stl, steals; Blk, blocks; To, turnovers; PF, personal fouls.

Table 1 and Figure 1 illustrate the ES and confidence intervals (95%CI) between home and away teams before and after COVID-19. Before COVID-19, home teams had a clear advantage over away games in terms of defensive rebounds (*p* < 0.001; ES, 0.21), assists (*p* < 0.001; ES, 0.21), two-point field goals made (*p* < 0.01; ES, 0.14), offensive rebounds (*p* < 0.01; ES, 0.12), and blocks (*p* < 0.05; ES, 0.11). By contrast, away teams missed more three-point field goals (*p* < 0.05; ES, 0.1), stole more (*p* < 0.05; ES, 0.1), and committed more personal fouls (*p* < 0.05; ES, 0.09) than home teams. In the ghost games after COVID-19, away teams secured more offensive rebounds than home teams which was the only indicator with statistical significance (*p* < 0.05; ES, 0.11).

**Table 1:** Descriptive statistics of match performances before and after COVID-19 in the NBA.

	Before COVID-19 (with spectators)				After COVID-19 (without spectators)			
	Home	Away	*p*-value	ES (95% CI)	Home	Away	*p*-value	ES (95% CI)
FT Made	17.67 ± 6.04	17.24 ± 5.74	0.097	−0.08 (−0.16, 0.01)	17.24 ± 5.88	16.9 ± 5.75	0.291	−0.06 (−0.18, 0.05)
FT Missed	5.25 ± 2.57	5.13 ± 2.71	0.294	−0.05 (−0.14, 0.04)	4.82 ± 2.69	4.76 ± 2.56	0.703	−0.02 (−0.14, 0.09)
FG2 Made	28.9 ± 5.3	28.15 ± 5.22	<0.01**	−0.14 (−0.23, −0.06)	28.31 ± 5.22	28.72 ± 5.24	0.167	0.08 (−0.03, 0.20)
FG2 Missed	25.86 ± 5.77	26.12 ± 5.81	0.324	0.04 (−0.04, 0.13)	25.11 ± 5.79	25.6 ± 5.69	0.147	0.09 (−0.03, 0.20)
FG3 Made	12.08 ± 3.84	12.01 ± 3.75	0.689	−0.02 (−0.11, 0.07)	12.86 ± 4.06	12.66 ± 4.12	0.404	−0.05 (−0.16, 0.07)
FG3 Missed	21.37 ± 5.06	21.87 ± 5.04	<0.05*	0.10 (0.01, 0.19)	21.93 ± 5.15	22 ± 5.13	0.797	0.02 (−0.10, 0.13)
OReb	10.28 ± 3.63	9.86 ± 3.6	<0.01**	−0.12 (−0.21, −0.03)	9.65 ± 3.61	10.05 ± 3.42	<0.05*	0.11 (0.00, 0.23)
DReb	35.01 ± 5.09	33.93 ± 5.39	<0.001***	−0.21 (−0.30, −0.12)	34.54 ± 4.88	34.56 ± 5.27	0.972	0.00 (−0.11, 0.12)
Ast	24.72 ± 4.92	23.7 ± 4.89	<0.001***	−0.21 (−0.30, −0.12)	25.04 ± 4.95	24.82 ± 4.87	0.473	−0.04 (−0.16, 0.07)
Stl	7.44 ± 2.82	7.72 ± 2.9	<0.05*	0.10 (0.01, 0.19)	7.49 ± 2.94	7.6 ± 2.88	0.551	0.04 (−0.08, 0.15)
Blk	5.05 ± 2.55	4.77 ± 2.41	<0.05*	−0.11 (−0.20, −0.02)	4.89 ± 2.45	4.8 ± 2.34	0.531	−0.04 (−0.15, 0.08)
To	14.46 ± 3.95	14.31 ± 3.93	0.423	−0.04 (−0.13, 0.05)	14.22 ± 3.8	13.95 ± 3.77	0.211	−0.07 (−0.19, 0.04)
PF	20.2 ± 4	20.57 ± 4.14	<0.05*	0.09 (0.00, 0.18)	19.55 ± 3.91	19.62 ± 4.08	0.757	0.02 (−0.10, 0.13)

*p < 0.05; **p < 0.01; ***p < 0.001. FT, free-throw; FG2, two field-goals; FG3, three field-goals; OReb, offensive rebounds; DReb, defensive rebounds; Ast, assists; Stl, steals; Blk, blocks; To, turnovers; PF, personal fouls.

### The key factors determined between winning and losing matches before and after COVID-19

**Figure 2:**
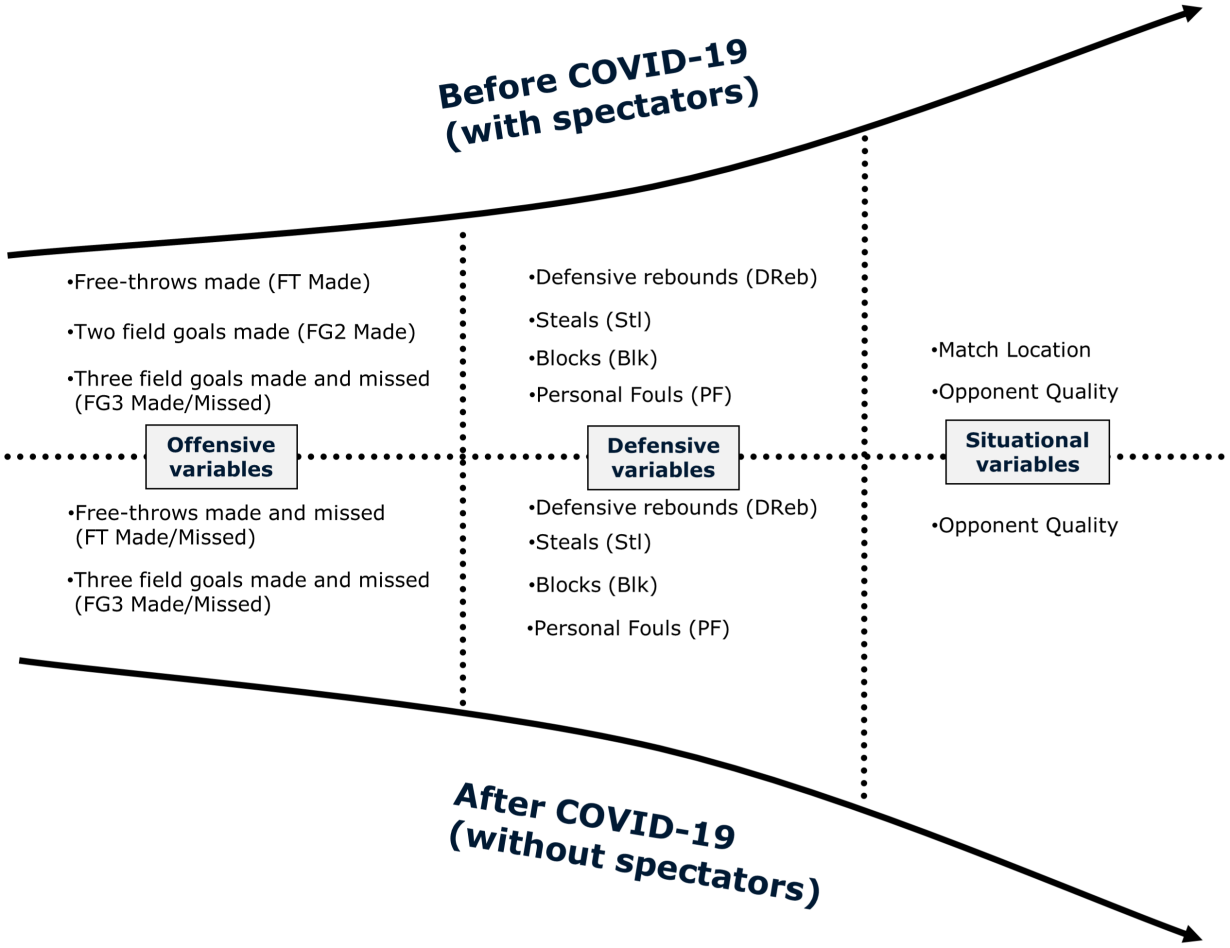
The key factors determined between winning and losing matches before and after COVID-19.

The inclusion of these 16 variables in a univariate binary logistic regression model resulted in 10 variables that were independently statistically significant winning factors before COVID-19. These variables were further analyzed by multivariable analysis (Table 2; Figure 2). After multivariable analysis, these 10 variables were still statistically significant which included free-throws made (OR, 1.24; 95%CI, 1.21–1.28; *p* < 0.001), two-point field goals made (OR, 1.44; 95%CI, 1.38–1.51; *p* < 0.001), both three-point field goals made (OR, 1.75; 95%CI, 1.64–1.86; *p* < 0.001) and missed (OR, 0.95; 95%CI, 0.92–0.98; *p* < 0.01), defensive rebounds (OR, 1.43; 95%CI, 1.37–1.49; *p* < 0.001), steals (OR, 1.46; 95%CI, 1.38–1.55; *p* < 0.001), blocks (OR, 1.14; 95%CI, 1.07–1.21; *p* < 0.001), personal fouls (OR, 0.95; 95%CI, 0.92–0.98; *p* < 0.01), match location (OR, 1.34; 95%CI, 1.02–1.78; *p* < 0.05), and opponent quality (OR, 2.35; 95%CI, 1.77–3.14; *p* < 0.001).

**Table 2:** Results relating to the logistic regression models run (dependent variable is “match outcome = WIN”).

	Before COVID-19 (with spectators)				AFTER (without spectators)			
	Univariate analysis		Multivariable analysis		Univariate analysis		Multivariable analysis	
	*p*-value	OR (95%CI)	*p*-value	OR (95%CI)	*p*-value	OR (95%CI)	*p*-value	OR (95%CI)
FT made	<0.001****	1.18 (1.09, 1.28)	<0.001****	1.24 (1.21, 1.28)	<0.01***	1.16 (1.05, 1.28)	<0.001****	1.12 (1.08, 1.15)
FT missed	0.450	0.97 (0.88, 1.06)			<0.1*	0.90 (0.80, 1.02)	<0.01***	0.91 (0.86, 0.97)
FG2 made	<0.01***	1.30 (1.10, 1.55)	<0.001****	1.44 (1.38, 1.51)	0.120	1.18 (0.96, 1.46)		
FG2 missed	0.112	0.87 (0.74, 1.03)			0.106	0.84 (0.68, 1.04)		
FG3 made	<0.001****	1.58 (1.32, 1.88)	<0.001****	1.75 (1.64, 1.86)	<0.01***	1.38 (1.11, 1.72)	<0.001****	1.27 (1.22, 1.33)
FG3 missed	<0.1*	0.85 (0.71, 1.00)	<0.01***	0.95 (0.92, 0.98)	<0.05**	0.80 (0.65, 0.99)	<0.001****	0.86 (0.83, 0.89)
OReb	0.108	1.15 (0.97, 1.37)			0.120	1.19 (0.96, 1.47)		
DReb	<0.001****	1.42 (1.37, 1.48)	<0.001****	1.43 (1.37, 1.49)	<0.001****	1.40 (1.33, 1.47)	<0.001****	1.28 (1.24, 1.33)
Ast	0.486	0.99 (0.95, 1.02)			0.148	1.03 (0.99, 1.08)		
Stl	<0.001****	1.46 (1.37, 1.55)	<0.001****	1.46 (1.38, 1.55)	<0.001****	1.43 (1.33, 1.54)	<0.001****	1.31 (1.24, 1.39)
Blk	<0.001****	1.13 (1.07, 1.20)	<0.001****	1.14 (1.07, 1.21)	<0.001****	1.14 (1.06, 1.23)	<0.05**	1.08 (1.01, 1.15)
To	0.430	0.93 (0.79, 1.10)			0.156	0.86 (0.69, 1.06)		
PF	<0.001****	0.94 (0.91, 0.97)	<0.01***	0.95 (0.92, 0.98)	<0.01***	0.94 (0.90, 0.99)	<0.01***	0.94 (0.91, 0.98)
Location	<0.1*	1.31 (0.99, 1.74)	<0.05**	1.34 (1.02, 1.78)	0.647	1.08 (0.77, 1.53)		
Opponent	<0.001****	2.39 (1.79, 3.19)	<0.001****	2.35 (1.77, 3.14)	<0.001****	1.99 (1.38, 2.87)	<0.001****	2.52 (1.84, 3.36)
Competition	0.841	0.95 (0.57, 1.57)			0.616	0.88 (0.55, 1.43)		

*p < 0.1; **p < 0.05; ***p < 0.01; ****p < 0.001. FT, free-throw; FG2, two field-goals; FG3, three field-goals; OReb, offensive rebounds; DReb, defensive rebounds; Ast, assists; Stl, steals; Blk, blocks; To, turnovers; PF, personal fouls; Location (the reference group is away games); Opponent (the reference group is the strong teams); Competition (the reference group is balanced competitions).

After the epidemic, nine variables were independently statistically significant winning factors by univariate binary logistic regression model and still statistically significant after multivariable analysis. These variables included both free-throws made (OR, 1.12; 95%CI, 1.08–1.15; *p* < 0.001) and missed (OR, 0.91; 95%CI, 0.86–0.97; *p* < 0.01), both three-point field goals made (OR, 1.27; 95%CI, 1.22–1.33; *p* < 0.001) and missed (OR, 0.86; 95%CI, 0.83–0.89; *p* < 0.001), defensive rebounds (OR, 1.28; 95%CI, 1.24–1.33; *p* < 0.001), steals (OR, 1.31; 95%CI, 1.24–1.39; *p* < 0.001), blocks (OR, 1.08; 95%CI, 1.01–1.15; *p* < 0.05), personal fouls (OR, 0.94; 95%CI, 0.91–0.98; *p* < 0.01), and opponent quality (OR, 2.52; 95%CI, 1.84–3.36; *p* < 0.001).

## Discussion

The aim of this study was to compare differences in the match performances during pre- and post-COVID-19 lockdown and to identify the key factors to ultimate success between matches with and without spectators. First, our study found that offensive rebounds were the only indicator differentiating between home and away games after the COVID-19 lockdown. Second, the game location was a key factor differentiating between winning and losing games before the COVID-19 lockdown, whereas it failed to be highlighted after the COVID-19 lockdown. Therefore, crowd support may have a significant impact on winning games in the NBA. Third, free-throws made, three-point field goals made, defensive rebounds, assists, steals, personal fouls, and opponent quality are in line with the previous studies (Ibáñez et al., 2003; Zhang et al., 2017) suggesting that key factors discriminated between win and loss whatever the period (pre- and post-COVID-19 lockdown) of analysis.

### The differences between home and away matches before and after COVID-19

Our study about the differences in match performances between home and away games before the COVID-19 pandemic is in line with the previous studies. Ehrlich and Potter (2022) found that the presence of fans matters to home team performance; in fact, “ghost games” eliminated HA in totality. In particular, Leicht et al. (2017) identified that home teams displayed better performance in terms of shooting efficiency, and offensive and defensive rebounds whereas away teams often make fouls to disturb the game pace of home teams and attempted more aggressive techniques such as steals combined with long-distance shooting to overcome game unexpected factors (e.g., dynamic tactics from home teams, self-negative psychological and behavioral states, crowd pressure, or less protection by the referees). After the COVID-19 pandemic, our study only highlighted offensive rebounds that discriminated between home and away games. Indeed, consistent with prior research (White and Sheldon, 2014), crowd attendance was associated with an improvement in home team rebounding differential (a measurement of effort). Rebounds are widely considered as a “hustle” and “grunt work” statistic since it requires players to fight for optimal position, where rough and physical contact is inevitable (Maheswaran et al., 2012). Offensive rebounds, which means to secure their own missed shot attempts, are considered a particularly robust measurement of effort because offensive players are often further from the rim when a shot is attempted, and they have a lower probability of securing the ball. Additionally, a substantial increase in attention both by the performer i.e., heightened self-focus as well as others at home in view (i.e., players, coaches, referees, and primarily the crowd), places a significant psychological inspiration on the performer for securing offensive rebounds.

### The key factors determined between winning and losing matches before and after COVID-19

The impact of game-related statistics on match outcome did not change much before and after COVID-19 according to the logistic regression models. Specifically, the field goal made, defensive rebounds, steals, and blocks were positively correlated with the winning games whereas the missed free-throws, missed three-point field goals, and personal fouls were negatively correlated with winning games. The highlighted positive variables were supported by Sampaio et al. (2010) who suggested that maintaining high shooting efficiency in offense and preventing a team from scoring with defensive pressure (e.g., steals, defensive rebounds, blocks) in defense can be a key determinant of the success of a team. In addition, Gómez et al. (2013b) and Paulauskas et al. (2018) noted that home teams perform better in terms of the mentioned positive variables than away teams. These studies speculated that crowd support was deemed to be critical, due to the spectators’ proximity to the playing area and the more constant, loud, inspiring sounds from the crowd, where enthusiastic cheers and chants can inspire initiative, and aggressiveness and encourage home players to try harder. However, our study found that these variables are still key factors associated with match outcome when playing without spectators. Therefore, the team should build up an effective of playing style to win basketball matches, whether or not crowds support matters. Outside players are required to have perimeter shooting skills, including three-point shooting, as well as to guard the opposition with aggressive pressure on the perimeter while inside players can prevent shooting from opponents and secure more defensive rebounds to organize fast breaks (Zhang et al., 2019b). The recent emergence of “small-ball” appears to be a critical factor in the NBA, as this style was more common in dominant teams during the “current” evolution of the NBA (Zhang et al., 2019a). In addition, our study also mentioned coaches who pay more attention to free throws when playing without crowds, especially for away teams, should seize the opportunity to improve the free-throw efficiency without being disturbed by home fans (Sampaio et al., 2006). It is worth noting that game location is the key factor determining between win and loss before the COVID-19 lockdown whereas it failed to be highlighted after the COVID-19 lockdown. Thus, this result appears to identify crowd support plays a key role in winning matches in the NBA which is in line with the previous studies (Huyghe et al., 2021) found that crowd support leads to HA in the NBA is a well-documented phenomenon that has been identified in over 7,000 games spanning 14 seasons (2004–2018) altogether.

There are limitations in the current study that should be considered. Our study only takes advantage of the natural experiment to consider the impact of crowd support on match performances, but match performances may be affected by referee bias, coaches’ tactics, and travel fatigue, so future studies are recommended to consider the interactive effect of these factors based on the current study. Additionally, the selected variables about match performance for our study were only based on traditional statistics, limiting to explanation of the key factors differentiating between win and loss during the period of pre-and-post COVID-19. A possible solution is to utilize each quarter’s data integrated with tracking and event data to make a spatio-temporal analysis to explore the impact of space control on match performance.

## Conclusion

In this study, researchers presented findings from a natural experiment caused by the COVID-19 to examine HA and its drivers and mechanisms in the NBA, especially the factor of crowd support. Our study found that offensive rebounds were the only indicator that presented the difference between home and away games after the COVID-19 lockdown. Second, the match location was the key factor determining between win and loss before the COVID-19 lockdown, whereas it failed to be highlighted after the COVID-19 lockdown. Free-throws made, three-point field goals made, defensive rebounds, assists, steals, personal fouls, and opponent quality were the common key factors discriminating between win and loss whatever pre -and post-COVID-19 lockdown.

Although only a descriptive case series design, our results offer some opinions that might be of interest to coaches and practitioners. Coaches may increase more practice in relation to the skills of box-out, thus allowing players to secure more offensive rebounds in the offense. Furthermore, coaches should adapt to the change in terms of HA by adjusting game strategies and player rotation to game success.

## Data availability statement

Publicly available datasets were analyzed in this study. This data can be found at: www.nba.com/stats/ and www.basketball-reference.com.

## Author contributions

PL, SZ, and MG contributed to the conception and design of the study. PL, XW, and SZ collected and organized the data. PL and JD performed the statistical analysis. PL wrote the first draft of the manuscript. All authors contributed to the article and approved the submitted version.

## Conflict of interest

The authors declare that the research was conducted in the absence of any commercial or financial relationships that could be construed as a potential conflict of interest.

## Publisher’s note

All claims expressed in this article are solely those of the authors and do not necessarily represent those of their affiliated organizations, or those of the publisher, the editors and the reviewers. Any product that may be evaluated in this article, or claim that may be made by its manufacturer, is not guaranteed or endorsed by the publisher.
